# Genome Sequence of *Corynebacterium pseudotuberculosis* Strain PA02 Isolated from an Ovine Host in the Amazon

**DOI:** 10.1128/genomeA.00838-16

**Published:** 2016-08-11

**Authors:** Gabriel R. S. Muge, Adonney A. O. Veras, Pablo H. C. G. de Sá, Ana Lídia Queiroz Cavalcante, Jorianne Thyeska Castro Alves, Ezequiel Morais, André G. M. Silva, Luís C. Guimarães, Vasco Azevedo, Adriana Ribeiro Carneiro Folador, Artur Silva, Rommel T. J. Ramos

**Affiliations:** a Center of Genomics and System Biology, Federal University of Pará (UFPA), Belém, Pará, Brazil; b Institute of Biological Sciences, Federal University of Minas Gerais (UFMG), Belo Horizonte, Minas Gerais, Brazil; c Institute of Veterinary Medicine, Federal University of Pará (UFPA), Campus of Castanhal, Pará, Brazil

## Abstract

In this work, we report the complete genome sequence of *Corynebacterium pseudotuberculosis* strain PA02 isolated from an ovine host. The genome contains 2,328,435 bp, a 52.2% G+C content, 2,035 coding sequences, 12 rRNA operons, 45 tRNAs, and 14 predicted pseudogenes.

## GENOME ANNOUNCEMENT

*Corynebacterium pseudotuberculosis* is a Gram-positive, facultative intracellular, pleomorphic, nonsporulating, noncapsulated, nonmotile pathogen (1–3). It is the causative agent of caseous lymphadenitis (CLA) in small ruminants, which is found in all of the world’s major sheep and goat herd areas and is characterized by the presence of caseous necrosis on the lymphatic glands or abscess formation in superficial lymph nodes and subcutaneous tissues. *C. pseudotuberculosis* also causes pyogranulomatous reactions, ulcerative lymphangitis, as well as mastitis and necrotic and ulcerative dermatitis in cattle (diseases with medical and veterinary relevance) (4).

CLA is a widespread disease that has been reported in many countries, including Australia, Brazil, Canada, New Zealand, South Africa, and the United States (4, 5). CLA leads to economic losses for sheep and goat farmers by causing skin deterioration and reducing yields of milk and wool. Moreover, *C. pseudotuberculosis* causes a visceral form of the disease that can affect internal organs, causing weight loss, death, and carcass condemnation (4).

*C. pseudotuberculosis* may be called the “perfect parasite” due to its ability to evade the immune system with apparent ease, once established within the host. *C. pseudotuberculosis* has a gene set repertoire for long-term survival outside a host environment (1).

*C. pseudotuberculosis* strain PA02 was isolated from an ovine host in Pará, Brazil. The genome sequencing was performed by Ion Torrent Personal Genome Machine (PGM) platform (Thermo, Fisher) using a fragment library, which produced a total of 392,463,062 bp representing a coverage of 171×. The FastQC version 0.11.4 software (http://www.bioinformatics.babraham.ac.uk/projects/fastqc) was used for assessing the quality of the raw data, and the FASTX-Toolkit (http://hannonlab.cshl.edu/fastx_toolkit) was used to trim and discard the reads with a Phred quality score below 20. The SPAdes genome assembler software (6) was used to generate 8 contigs, with an *N*_50_ of 713,155 bp, a maximum contig length of 819,241 bp, and a total size of 2,328,435 bp. The automatic genome annotation was performed using RAST version 2.0 (7). tRNAs and rRNAs were predicted using tRNAScan-SE version 1.12 (8) and RNAmmer version 1.2 (9) software, respectively.

The manual curation of the annotation was performed through CLC Genomics Workbench version 8, Artemis version 16.0.0 (10), and the UniProt (http://www.uniprot.org) and NCBI nonredundant (https://www.ncbi.nlm.nih.gov) databases.

The *C. pseudotuberculosis* strain PA02 genome consists of a circular chromosome of 2,328,435 bp, with 52.2% G+C content, 2,035 coding sequences, 12 rRNAs, 45 tRNAs, and 14 pseudogenes.

### Accession number(s).

The *C. pseudotuberculosis* PA02 whole-genome shotgun project has the project accession number PRJNA318798. This version used for this project has the accession number CP015309.
